# Subjective social status and mortality: the English Longitudinal Study of Ageing

**DOI:** 10.1007/s10654-018-0410-z

**Published:** 2018-05-19

**Authors:** Panayotes Demakakos, Jane P. Biddulph, Cesar de Oliveira, Georgios Tsakos, Michael G. Marmot

**Affiliations:** Department of Epidemiology and Public Health, University College London, London, UK

**Keywords:** Ageing, Inequalities, Mortality, Social status, Socioeconomic position

## Abstract

Self-perceptions of own social position are potentially a key aspect of socioeconomic inequalities in health, but their association with mortality remains poorly understood. We examined whether subjective social status (SSS), a measure of the self-perceived element of social position, was associated with mortality and its role in the associations between objective socioeconomic position (SEP) measures and mortality. We used Cox regression to model the associations between SSS, objective SEP measures and mortality in a sample of 9972 people aged ≥ 50 years from the English Longitudinal Study of Ageing over a 10-year follow-up (2002–2013). Our findings indicate that SSS was associated with all-cause, cardiovascular, cancer and other mortality. A unit decrease in the 10-point continuous SSS measure increased by 24 and 8% the mortality risk of people aged 50–64 and ≥ 65 years, respectively, after adjustment for age, sex and marital status. The respective estimates for cardiovascular mortality were 36 and 11%. Adjustment for all covariates fully explained the association between SSS and cancer mortality, and partially the remaining associations. In people aged 50–64 years, SSS mediated to a varying extent the associations between objective SEP measures and all-cause mortality. In people aged ≥ 65 years, SSS mediated to a lesser extent these associations, and to some extent was associated with mortality independent of objective SEP measures. Nevertheless, in both age groups, wealth partially explained the association between SSS and mortality. In conclusion, SSS is a strong predictor of mortality at older ages, but its role in socioeconomic inequalities in mortality appears to be complex.

**Electronic supplementary material:**

The online version of this article (10.1007/s10654-018-0410-z) contains supplementary material, which is available to authorized users.

## Introduction

People’s position in the social hierarchy is strongly linked to health in a graded way; the higher the position the better the health. The resulting socioeconomic inequalities in health, the social gradient in health, have been widely observed [1–8]. The burden associated with socioeconomic inequalities is immense as each year millions of deaths and years of potential life lost across the world are attributed to the unequal distribution of social and economic resources and its individual, community and societal implications [9, 10]. Research has focused on explaining socioeconomic inequalities in health and identifying causal pathways that might constitute targets for prevention [10]. Various explanations have been put forward about what might explain the graded association between socioeconomic position (SEP) and risk of ill-health and death [11–18], while empirical research has offered evidence on many different mediating factors ranging from unhealthy behaviours to health insurance and from control over life to work stress [1, 10, 19–21].

Subjective social status (SSS), a concept that refers to self-perceptions of one’s own social position, has received less attention in epidemiological research and its role in socioeconomic inequalities in health remains poorly understood. This is despite its potential to add to the current understanding of socioeconomic inequalities in health when used in conjunction with conventional SEP measures. SSS is a measure of SEP as it is perceived by the individuals themselves; one’s personal translation of objective SEP. Thus, it is a measure of SEP as experienced and internalised by individuals and for that reason it is expected to be closely related to health and a series of personal attributes including behaviours, attitudes, values and worldviews. Further, SSS captures personal individualised aspects of one’s social identity and socioeconomic position [22] such as lifetime achievement and recognition by others, prestige and a successful family life that conventional SEP measures do not [23]. For that reason its use in epidemiological research can broaden our ability to understand socioeconomic inequality beyond conventional SEP measures. In addition, unlike commonly used measures that tap into specific SEP dimensions, SSS is a summary measure of SEP that is easy to measure and thus appealing to survey designers.

Previous research has used SSS to predict various health outcomes [23–26], but paradoxically SSS has only rarely been used to predict mortality [27]. At the moment it remains unclear how strongly SSS is associated with mortality and what is its role in the associations between objective SEP measures and mortality. We aimed to cover this gap in the literature by examining whether and how SSS might be associated with mortality at older ages. To provide a fuller picture of this association we examined both all-cause and cause-specific mortality. Because evidence suggests that SSS might partially mediate the associations between objective SEP measures and different health outcomes, we also explored whether SSS mediated the associations between paternal occupational class when respondents were 14 years old, education, occupational class, income, wealth and mortality. The broad age range of our sample, that is ≥ 50 years, allowed for an exploration of age differences in the association between SSS and mortality that can substantially add to the limited literature on socioeconomic inequalities in health in old age [28].

## Methods

### Participants

The English Longitudinal Study of Ageing (ELSA) is a prospective observational study of community-dwellers aged ≥ 50 years that was designed to be nationally representative. At baseline, in 2002–2003, the ELSA sample comprised 11,391 individuals who previously had participated in the Health Survey for England. The Health Survey for England is a national health examination survey, which each year recruits a different nationally representative sample using a stratified probability design. ELSA has been approved by the National Research Ethics Service and informed consent has been obtained by the participants. More details about ELSA can be found at: http://www.elsa-project.ac.uk/. Our analytical sample included 9972 ELSA participants after the exclusion of 362 participants with proxy or partial interviews, 464 participants without valid mortality data (most of whom did not consent to link their interview data with the mortality records), 335 participants who did not respond to the SSS question and were assumed to be missing not at random and 258 participants with missing values in covariates (excluding BMI).

### Mortality

We used mortality data from the Office for National Statistics that spanned a period of ten years, from the date of the baseline interview in 2002–2003 to February 2013. Deaths were classified according to International Classification of Diseases (ICD) 10th Edition. Deaths with ICD10 codes C00 to C97 were classified as cancer deaths and those with ICD10 codes I00 to I99 as cardiovascular deaths. All remaining deaths were classified as other.

### Subjective Social Status

We measured baseline SSS, one’s perceptions of own social position, using a drawing of a ladder with 10 rungs [29]. Participants were asked to place themselves on one of the ten rungs after they were primed to think of the ladder as a representation of society with the use of the following vignette: ‘Think of this ladder as representing where people stand in our society. At the top of the ladder are the people who are the best off—those who have the most money, most education and best jobs. At the bottom are the people who are the worst off—who have the least money, least education, and the worst jobs or no jobs. The higher up you are on this ladder, the closer you are to the people at the very top and the lower you are, the closer you are to the people at the very bottom. Please mark a cross on the rung on the ladder where you would place yourself’. Respondents who had put their mark in between two rungs were assigned to the higher of these rungs.

We used the reversed ladder score as a continuous measure with a value range from 1 to 10 with higher values denoting lower SSS. The distribution of the non-reversed ladder score by age along with descriptive statistics are presented in the online Appendix (Figures S1 and S2 and Table S1).

### Covariates

We measured the following indicators of objective SEP: paternal/main carer’s occupational class when respondents aged 14 years, education, occupational class, income and wealth. Paternal/main carer’s occupational class at age 14 years was measured using a 4-category variable (managerial and professional occupations including running own business, intermediate occupations mostly services workers, routine occupations such as plant workers including a small number of unemployed and disabled, other including those in the armed forces). Education was measured using a 3-category educational attainment variable (A-level or higher, O-level/secondary education, no educational qualifications). Occupational class was measured using the National Statistics Socio-economic Classification (managerial and professional occupations, intermediate occupations, semi-routine and routine occupations, other including those who never worked). Tertiles of equivalised weekly household income and total net non-pension household wealth were used to measure income and wealth, respectively. Age, sex, marital status, unhealthy behaviours (smoking and physical activity including participation in sports, leisure activities and household chores), obesity (BMI categories), and elevated depressive symptoms (defined using the cut point of ≥ 4 symptoms on the 8-item CES-D, which corresponds to the cut point of ≥ 16 on the full 20-item CES-D [30] that has been widely used to identify possible cases of depression) were also measured as covariates. All these covariates were measured at baseline in 2002–2003, except for BMI, which was measured at ELSA wave 2, in 2004–2005. BMI was also the only adjustment variable for which we imputed missing values (n = 945). We did that to avoid the unnecessary exclusion of a large number of participants from the analyses.

### Statistical analysis

We examined differences in SSS by the baseline characteristics of the sample. We estimated Cox proportional hazard regression models of the associations between SSS and all-cause and cause-specific mortality. We checked and confirmed that the proportional hazards assumption was met using the Schoenfeld residuals test and log–log plots of survival on a categorical SSS variable. Time-to-event (in months) was calculated as the time that elapsed from the date of the baseline interview in 2002–2003 to the date of death or censoring (for consenting participants not known to be dead by the end of the study, the censoring date was February 2013). We first estimated the unadjusted models, which we adjusted for age, sex, and marital status, then for smoking, physical activity and BMI, and finally for elevated depressive symptoms. We also estimated a series of models of the association between SSS and all-cause mortality that were initially adjusted for age, sex and marital status and then for each of the objective SEP measures. We assumed that SSS, a measure of self-perceived SEP, is a good candidate mediator of the association between objective SEP and mortality. We examined this assumption using a common mediation approach that concentrated on the change in the association of interest after adjustment for the mediating variable. We estimated models for each objective SEP measure and its association with all-cause mortality, which were initially adjusted for age, sex and marital status, and then for SSS. Because interaction analyses indicated that the association between SSS and mortality varied by age but not sex, we stratified all analyses by age using the cut point of 65 years as described elsewhere [1]. Despite excluding from analyses participants who avoided to respond to the SSS question (N = 335), there was still a number of participants (N = 660) with missing SSS values, who did not complete the whole pen-and-paper questionnaire that contained the SSS question, not just the SSS question. We assumed that SSS values for these participants were missing at random. Based on this assumption and to minimise non-response bias, we imputed missing SSS values for these participants using chained equations in STATA 14. The imputation model included the covariates included in the analysis model, a retirement status variable, which was a strong predictor of SSS in our data, the mortality variable and the Nelson–Aalen estimate of the cumulative hazard to the survival time[31].

For comparison reasons and to ascertain that the imputed data are comparable to the observed data, we performed additional analyses where we estimated models using only the observed data (Table S2 in the online supplement).

## Results

In both age groups, male, married, non-smokers, non-obese and physically active participants as well as those who did not report elevated depressive symptoms on average scored higher on the SSS scale (Table 1). As expected, there were strong positive associations between SSS and measures of objective SEP. The wealth differences in SSS score were the greatest observed in our data. In the younger age group, there was difference of 2 points between participants in the highest and lowest wealth tertile, while in the older age group this difference was 1.7 points.

**Table 1:** The baseline characteristics of the sample by age, English Longitudinal Study of Ageing 2002–2013

	Age group: 50–64 years			Age group: ≥ 65 years		
	N (%)^a^	Mean SSS^b^ (95% CI)	*P* value^c^	N (%)^a^	Mean SSS^b^ (95% CI)	*P* value^c^
**N**	5275	–	–	4697	–	–
*Mean age (95% CI)*	56.8 (56.7–56.9)	–	–	73.8 (73.6–73.9)	–	–
*Sex*			< 0.001			0.010
Male	2454 (46.5)	6.0 (5.9–6.1)		2138 (45.5)	5.6 (5.5–5.7)	
Female	2821 (53.5)	5.8 (5.7–5.8)		2559 (54.5)	5.5 (5.4–5.5)	
*Marital status*			< 0.001			< 0.001
Married	3949 (74.9)	6.0 (6.0–6.1)		2738 (53.8)	5.7 (5.6–5.8)	
Other	1326 (25.1)	5.4 (5.2–5.5)		1959 (41.7)	5.3 (5.2–5.4)	
*Smoking*			< 0.001			< 0.001
Current smoker	1189 (22.5)	5.3 (5.2–5.4)		587 (12.5)	4.9 (4.8–5.1)	
Former smoker	2191 (41.5)	6.0 (6.0–6.1)		2463 (52.4)	5.6 (5.5–5.7)	
Never smoker	1895 (35.9)	6.1 (6.0–6.1)		1647 (35.1)	5.6 (5.6–5.7)	
*Physical activity at least once a week*			< 0.001			< 0.001
Vigorous-intensity	1798 (34.1)	6.3 (6.2–6.4)		964 (20.5)	5.9 (5.8–6.0)	
Moderate-intensity	2552 (48.4)	5.9 (5.8–6.0)		2224 (47.3)	5.6 (5.6–5.7)	
Mild-intensity	597 (11.3)	5.2 (5.0–5.3)		827 (17.6)	5.2 (5.1–5.4)	
Physically inactive	328 (6.2)	4.7 (4.5–4.9)		682 (14.5	5.0 (4.8–5.1)	
*Body mass index* ^d^			< 0.001			0.003
< 25 kg/m^2^	1493 (28.3)	6.0 (5.9–6.1)		1195 (25.5)	5.5 (5.4–5.6)	
25 to < 30 kg/m^2^	2154 (40.8)	6.0 (5.7–6.1)		1922 (40.9)	5.7 (5.7–5.7)	
≥ 30 kg/m2	1254 (23.8)	5.7 (5.6–5.8)		1053 (22.4)	5.4 (5.6–5.8)	
Missing	374 (7.1)	5.6 (5.4–5.8)		527 (11.2)	5.3 (5.2–5.5)	
*Elevated depressive symptoms*			< 0.001			< 0.001
No	4484 (85.0)	6.1 (6.0–6.1)		3907 (83.2)	5.7 (5.6–5.7)	
Yes	791 (15.0)	4.7 (4.5–4.8)		790 (16.8)	4.8 (4.7-5.0)	
*Education*			< 0.001			< 0.001
A-level or higher	1910 (36.2)	6.6 (6.5–6.7)		944 (20.1)	6.5 (6.4–6.6)	
GCSE/O-level/other qualification	1721 (32.6)	5.8 (5.7–5.9)		1244 (26.5)	5.7 (5.6–5.8)	
No educational qualifications	1644 (31.2)	5.1 (5.0–5.2)		2509 (53.4)	5.1 (5.0–5.2)	
*Occupational class* ^e^			< 0.001			< 0.001
Managerial and professional occupations	1748 (33.1)	6.7 (6.6–6.8)		1215 (25.9)	6.3 (6.2–6.4)	
Intermediate occupations	1231 (23.3)	6.0 (5.9–6.1)		1110 (23.6)	5.7 (5.6–5.8)	
Semi-routine and routine occupations	2246 (42.6)	5.2 (5.1–5.3)		2269 (48.3)	5.1 (5.0–5.2)	
Other/never worked	50 (1.0)	5.2 (4.6–5.9)		103 (2.2)	5.5 (5.1–6.0)	
*Paternal/carer’s occupational class when respondent was 14* *years old*^e^			< 0.001			< 0.001
Managerial and professional occupations/run own business	1617 (30.7)	6.4 (6.3–6.5)		1204 (25.6)	6.1 (6.0–6.2)	
Intermediate occupations	1700 (32.2)	5.8 (5.8–5.9)		1509 (32.1)	5.5 (5.4–5.6)	
Routine occupations/casual jobs/unemployed/disabled	1764 (33.4)	5.4 (5.4–5.5)		1689 (36.0)	5.2 (5.1–5.3)	
Other (incl. Armed Forces)	194 (3.7)	5.9 (5.6–6.2)		295 (6.3)	5.4 (5.2–5.7)	
*Weekly household income tertiles*			< 0.001			< 0.001
Highest (≥ £262.79)	2453 (46.5)	6.5 (6.4–6.6)		994 (21.2)	6.6 (6.5–6.7)	
Middle (< £262.79 to ≥ £155.19)	1616 (30.6)	5.6 (5.6–5.7)		1714 (36.5)	5.6 (5.5–5.7)	
Lowest (< £155.19)	1206 (22.9)	4.9 (4.8–5.0)		1989 (42.3)	5.0 (4.9–5.0)	
*Total net non-pension household wealth tertiles*			< 0.001			< 0.001
Highest (≥ £203,000)	1981 (37.6)	6.7 (6.7–6.8)		1392 (29.7)	6.5 (6.4–6.5)	
Middle (< £203,000 to ≥ £76,020)	1821 (34.5)	5.9 (5.8–6.0)		1552 (33.2)	5.5 (5.4–5.6)	
Lowest (< £76,020)	1473 (27.9)	4.7 (4.6–4.8)		1735 (37.1)	4.8 (4.8–4.9)	

*CI* confidence interval, *SSS* subjective social status

^a^Unless stated otherwise

^b^To facilitate understanding, SSS has not been reversed in this table. Higher values denote higher SSS

^c^*P* values were calculated using the observed (non-imputed) data and the analysis of variance test

^d^The observed (non-imputed) BMI data were used. The “Missing” category was not used in the calculation of the *P* value

^e^The “Other” category was not used in the calculation of the *P* value

We observed 402 and 1861 deaths in the younger and older age groups, respectively (Table 2). In the younger age group, all-cause mortality risk increased by 24% per unit increase in the SSS score after adjustment for age, sex, and marital status, while in the older age group, this increase was smaller at 8%. SSS appeared to be associated more strongly with CVD-related and other mortality than with cancer-related mortality. As in all-cause mortality, these associations were stronger in the younger age group compared with older age group. Adjustments for unhealthy behaviours, BMI and elevated depressive symptoms fully explained the association between SSS and other (in participants aged ≥ 65 years) and cancer mortality and partially the associations between SSS and all-cause, CVD and other mortality (in those aged 50–64 years).

**Table 2:** The association between subjective social status and all-cause and cause-specific mortality by age, English Longitudinal Study of Ageing 2002–2013

	Age group: 50–64 years	Age group: ≥ 65 years
*All-cause mortality*		
No of deaths	402	1861
Deaths/1000 person years	7.5 (6.8–8.3)	46.3 (44.2–48.6)
Model 1 HR (95% CI)	1.25 (1.18–1.31)	1.08 (1.06–1.11)
Model 2 HR (95% CI)	1.24 (1.18–1.31)	1.08 (1.05–1.11)
Model 3 HR (95% CI)	1.14 (1.07–1.20)	1.04 (1.01–1.06)
Model 4 HR (95% CI)	1.11 (1.05–1.18)	1.03 (1.00–1.06)
*Cardiovascular mortality*		
No of deaths	99	663
Deaths/1000 person years	1.9 (1.5–2.3)	16.5 (15.3–17.8)
Model 1 HR (95% CI)	1.36 (1.22–1.51)	1.11 (1.06–1.17)
Model 2 HR (95% CI)	1.36 (1.22–1.51)	1.11 (1.05–1.16)
Model 3 HR (95% CI)	1.18 (1.06–1.32)	1.07 (1.02–1.12)
Model 4 HR (95% CI)	1.15 (1.03–1.29)	1.06 (1.01–1.11)
*Cancer mortality*		
No of deaths	193	514
Deaths/1000 person years	3.6 (3.1–4.2)	12.8 (11.7–13.9)
Model 1 HR (95% CI)	1.14 (1.05–1.23)	1.06 (1.00–1.11)
Model 2 HR (95% CI)	1.13 (1.05–1.23)	1.06 (1.01–1.12)
Model 3 HR (95% CI)	1.07 (0.98–1.16)	1.03 (0.98–1.09)
Model 4 HR (95% CI)	1.05 (0.97–1.15)	1.03 (0.97–1.08)
Other mortality		
No of deaths	110	684
Deaths/1000 person years	2.1 (1.7–2.5)	17.0 (15.8–18.3)
Model 1 HR (95% CI)	1.35 (1.22–1.49)	1.07 (1.03–1.12)
Model 2 HR (95% CI)	1.32 (1.20–1.46)	1.06 (1.01–1.11)
Model 3 HR (95% CI)	1.21 (1.09–1.34)	1.02 (0.97–1.07)
Model 4 HR (95% CI)	1.17 (1.05–1.31)	1.00 (0.96–1.05)
*Sample sizes*		
No of participants	5275	4697
Person years of follow-up	53431	40196

*CI* confidence interval, *HR* hazard ratio

Model 1 represents the unadjusted association

Model 2 is adjusted for age, sex, and marital status

Model 3 is adjusted for age, sex, marital status, smoking, physical activity, and BMI

Model 4 is adjusted for age, sex, marital status, smoking, physical activity, BMI and elevated depressive symptoms

Hazard ratios denote hazard change per unit decrease in SSS

In both age groups, the association between SSS and all-cause mortality was little affected by adjustment for most objective SEP measures, except for the adjustment for wealth, which explained a considerable part of it (Table 3). The associations between measures of objective SEP and all-cause mortality were partially explained, to a varying extent, after adjustment for SSS (Table 4). In the younger age group, SSS explained a large part of the associations between education and adult occupational class and all-cause mortality, and a smaller part of the associations between childhood occupational class, income and wealth and all-cause mortality. In the older age group, SSS explained a smaller part of these associations.

**Table 3:** The association between subjective social status and all-cause mortality by age, English Longitudinal Study of Ageing 2002–2013

	Age group: 50–64 years	Age group: ≥ 65 years
*All-cause mortality*		
No of deaths	402	1861
Deaths/1000 person years	7.5 (6.8–8.3)	46.3 (44.2–48.6)
Model 1 HR (95% CI)	1.24 (1.18–1.31)	1.08 (1.05–1.11)
Model 2 HR (95% CI)	1.22 (1.15–1.29)	1.06 (1.03–1.09)
Model 3 HR (95% CI)	1.21 (1.14–1.28)	1.06 (1.03–1.09)
Model 4 HR (95% CI)	1.22 (1.16–1.29)	1.07 (1.04–1.10)
Model 5 HR (95% CI)	1.19 (1.13–1.26)	1.06 (1.03–1.09)
Model 6 HR (95% CI)	1.14 (1.08–1.21)	1.04 (1.01–1.07)
*Sample sizes*		
No of participants	5275	4697
Person years of follow-up	53,431	40,196

*CI* confidence interval, *HR* hazard ratio

Model 1 is adjusted for age, sex, and marital status

Model 2 is adjusted for age, sex, marital status and education

Model 3 is adjusted for age, sex, marital status and occupational class

Model 4 is adjusted for age, sex, marital status and paternal/carer’s occupational class when respondent was 14 years old

Model 5 is adjusted for age, sex, marital status and equivalised weekly household income tertiles

Model 6 is adjusted for age, sex, marital status and total net non-pension household wealth tertiles

Hazard ratios denote hazard change per unit decrease in SSS

**Table 4:** The associations between each of the objective socioeconomic position measures and all-cause mortality by age, English Longitudinal Study of Ageing 2002–2013

Age group: 50–64 years			
	A-level or higher	O-level/GCSE	No qualifications
*Predictor: Education*			
No of deaths	121	103	178
No of participants	1910	1721	1644
Model 1 HR (95% CI)	1.00 (reference)	1.00 (0.77–1.31)	1.73 (1.36–2.18)
Model 2 HR (95% CI)	1.00 (reference)	0.86 (0.66–1.13)	1.28 (1.00–1.65)

	Managerial/professional	Intermediate	Semi-routine/routine
*Predictor: Occupational class* ^a^			
No of deaths	94	89	213
No of participants	1748	1231	2246
Model 1 HR (95% CI)	1.00 (reference)	1.46 (1.09–1.95)	1.79 (1.41–2.29)
Model 2 HR (95% CI)	1.00 (reference)	1.26 (0.94–1.69)	1.33 (1.03–1.73)

	Managerial/professional/run own business	Intermediate	Routine/casual/unemployed/disabled
*Predictor: Paternal/carer’s occupational class when respondent was 14* *years old*^b^			
No of deaths	85	139	162
No of participants	1617	1700	1764
Model 1 HR (95% CI)	1.00 (reference)	1.48 (1.13–1.94)	1.66 (1.27–2.15)
Model 2 HR (95% CI)	1.00 (reference)	1.30 (0.99–1.71)	1.36 (1.04–1.77)

	Highest	Intermediate	Lowest
*Predictor: Equivalised weekly household income tertiles*			
No of deaths	115	149	138
No of participants	2453	1616	1206
Model 1 HR (95% CI)	1.00 (reference)	1.85 (1.45–2.36)	2.20 (1.71–2.84)
Model 2 HR (95% CI)	1.00 (reference)	1.59 (1.24–2.05)	1.67 (1.27–2.19)

	Highest	Intermediate	Lowest
*Predictor: Total net non-pension household wealth tertiles*			
No of deaths	92	116	194
No of participants	1981	1821	1473
Model 1 HR (95% CI)	1.00 (reference)	1.43 (1.08–1.88)	3.01 (2.34–3.89)
Model 2 HR (95% CI)	1.00 (reference)	1.26 (0.96–1.67)	2.30 (1.73–3.06)

Age group: ≥ 65 years			
	A-level or higher	O-level/GCSE	No qualifications
Predictor: Education			
No of deaths	308	430	1123
No of participants	944	1244	2509
Model 1 HR (95% CI)	1.00 (reference)	1.07 (0.92–1.24)	1.33 (1.17–1.51)
Model 2 HR (95% CI)	1.00 (reference)	1.02 (0.88–1.18)	1.23 (1.08–1.40)

	Managerial/professional	Intermediate	Semi-routine/routine
*Predictor: Occupational class* ^a^			
No of deaths	441	404	965
No of participants	1215	1110	2269
Model 1 HR (95% CI)	1.00 (reference)	1.12 (0.98–1.29)	1.32 (1.18–1.48)
Model 2 HR (95% CI)	1.00 (reference)	1.09 (0.95–1.25)	1.25 (1.11–1.40)

	Managerial/professional/run own business	Intermediate	Routine/casual/unemployed/disabled
*Predictor: Paternal/carer’s occupational class when respondent was 14* *years old*^b^			
No of deaths	445	594	702
No of participants	1204	1509	1689
Model 1 HR (95% CI)	1.00 (reference)	1.14 (1.01–1.29)	1.25 (1.11–1.41)
Model 2 HR (95% CI)	1.00 (reference)	1.10 (0.97–1.24)	1.18 (1.05–1.33)

	Highest	Intermediate	Lowest
*Predictor: Equivalised weekly household income tertiles*			
No of deaths	292	658	911
No of participants	994	1714	1989
Model 1 HR (95% CI)	1.00 (reference)	1.25 (1.09–1.43)	1.34 (1.17–1.53)
Model 2 HR (95% CI)	1.00 (reference)	1.18 (1.02–1.36)	1.23 (1.06–1.41)

	Highest	Intermediate	Lowest
*Predictor: Total net non-pension household wealth tertiles*			
No of deaths	415	548	898
No of participants	1392	1552	1753
Model 1 HR (95% CI	1.00 (reference)	1.17 (1.03–1.33)	1.72 (1.53–1.95)
Model 2 HR (95% CI)	1.00 (reference)	1.13 (0.99–1.28)	1.63 (1.44–1.86)

*CI* confidence interval, *HR* hazard ratio

Model 1 is adjusted for age, sex, and marital status

Model 2 is adjusted for age, sex, marital status and subjective social status

Hazard ratios denote the differences in the hazards between the reference category and other categories of the predictor variable

^a^For clarity reasons, the HR for the small “Other/Never worked” category are not presented

^b^For clarity reasons, the HR for the small “Other” category are not presented

## Discussion

In a national sample of people aged ≥ 50 years, we found subjective social status, one’s perceptions of their own social status, to be inversely associated with all-cause and cause-specific mortality. These associations were stronger in participants aged 50–64 years compared with those aged ≥ 65 years and were explained to a varying extent by unhealthy behaviours, obesity and elevated depressive symptoms. SSS partially mediated the associations between objective SEP measures such as education and occupational class and mortality, especially in participants aged 50–64 years. SSS appears to explain a unique part of mortality that no single objective SEP measure could explain. Nevertheless, in both age groups, wealth partially explained the association between SSS and mortality; a strong indication that the association between SSS and mortality can partially be attributed to SSS reflecting one’s wealth and being a product of assets ownership and material deprivation.

Despite the importance of SSS to better understand socioeconomic inequalities in health and an expanding literature on its associations with morbidity [24–26, 32], very little research has focused on the association between SSS and mortality. We are aware of only one individual-level study on the association between SSS and mortality [27]. Their findings partially concur with ours; they examined separately men and women aged 40–65 years and found SSS to predict mortality over 3.5 years of follow-up in men, but not in women. Other studies have explored the associations between self-perceptions of specific dimensions of SEP such as self-perceived income and wealth [33, 34], relative deprivation [35, 36], occupational prestige [37], and perceptions about own work trajectory [38] and all-cause mortality. Notwithstanding methodological differences, our findings concur with those of most previous studies [34–38].

Our study has strengths and limitations that need to be acknowledged. The use of data from a survey that is designed to be nationally representative is a strength and makes our findings more generalizable to community-dwellers aged ≥ 50 years. The novelty of our findings should also be stressed. Our study is the first to examine the association between SSS and mortality in people aged ≥ 65 years and the first to examine the association between SSS and cause-specific mortality. It is also the first systematic attempt to explore the interrelationships between SSS and commonly used objective SEP measures in relation to mortality. Finally, the comprehensive assessment of SEP and the 10-year long follow-up make our study a thorough investigation of the association between SSS, SEP and mortality. A weakness of our study is our inability to fully control for non-response bias. We were able to impute missing at random SSS values and link almost all participant data with mortality records, but our sample remained to some extent selected as at baseline it included community-dwellers who have survived at least to age ≥ 50 years. Further, the baseline household response rate was very good at 70%, but nevertheless left some room for non-response bias. Another weakness of our study is its purely exploratory character. Our work neither proposed nor tested any theoretical model of the associations between objective SEP measures, SSS, and mortality. However, it generated basic evidence about these associations, which can then be used to build a well-defined testable model of socioeconomic inequalities in mortality. The mediation analysis presented in Table 4 is based on the conceptual argument that SSS is most likely a product of objective SEP and thus a good candidate mediator of the associations between each one of the objective SEP measures and all-cause mortality. Our approach was simple and based on a three-variable system with a single mediator, which is expected to be associated with both the predictor and the outcome and explain to a varying extent the effect of the predictor on the outcome [39]. This approach neither allows a simultaneous examination of direct and indirect effects nor fully accounts for confounding [40].

Our findings indicate that SEP has a substantive subjective dimension that is strongly related to all-cause mortality in three different ways. First, SSS mediates to a varying degree the associations between objective SEP measures and mortality. Second, SSS to some extent appears to be an independent predictor of mortality, possibly as a measure of facets of social position not captured by objective SEP measures. Third, SSS is partially associated with mortality as a product of wealth and material circumstances.

In people aged 50–64 years, SSS explained to a considerable extent the associations between objective SEP measures and mortality. On the basis that objective SEP is expected to shape people’s perceptions of their standing on the societal hierarchy and influence their social identity, our findings likely suggest that self-perceptions of own social status as captured by SSS is an important channel through which objective SEP exerts a considerable part of its effect on mortality. In people aged 50–64 years, SSS appears to be explaining to a greater extent the associations between education and adult social class and mortality. We can only speculate that this might happen because social comparisons among working age people are typically made on the basis of education and adult occupational class and thus these two SEP measures might be more important for the formation of perceptions of own social status, that is SSS, than other SEP measures in this age group. Further, education and childhood and adult social classes are in a sense historic SEP markers and thus expected to exert their impact on mortality mostly indirectly via more contemporary SEP measures such as SSS, income and wealth.

In people aged ≥ 65 years, SSS continues to be a significant predictor of mortality. Nevertheless, the importance of SSS as a mediator of the associations between SEP measures and mortality is somewhat decreased. This change in the role of SSS in socioeconomic inequalities in mortality likely can be attributed to its dynamic and age-dependant character. Past the age of 65 years, where most people are retirees and no longer financially active, SSS might be less about education and adult occupational class and more about more dimensions of social position that are perhaps more meaningful in this age group such as lifetime achievement, successfulness in family life, prestige and recognition within one’s local community. These more individualised dimensions of SEP can also be important for survival in old age because of their connection with the provision of key resources such as emotional support, care and practical help.

The observed age differences in the association between SSS and mortality are expected. It is known that the effect of most risk factors on mortality decreases with age partially as a result of survivor bias. Nevertheless, the public health importance of SSS inequalities in people aged ≥ 65 years should not be underestimated. Most deaths occur past the age of 65 years and that means that even small differences in the relative risk of mortality according to SSS in this age group correspond to great differences in the number of deaths.

Regarding specific causes of death, in accordance with previous evidence suggesting a inverse association between objective SEP measures and CVD [6], we found that SSS is strongly associated with CVD-related mortality in our participants. The strength and persistence of this association underline the importance of the subjective dimension of SEP for cardiovascular mortality. The same applies to the association between SSS and other mortality in participants aged 50–64 years, which is indicative of a strong association between the subjective aspects of SEP and death from respiratory and other causes including suicide and accidents. The association between SSS and cancer-related mortality was strong, especially among participants 50–64 years, but fully explained after adjustment for unhealthy behaviours and obesity.

### Conclusions and public health implications

In summary, our study provides substantial evidence for an inverse association between SSS and mortality. SSS appears to partially mediate the associations between objective SEP measures such as education and occupational class and mortality—especially in people aged 50–64 years. To some extent SSS appears to be associated with mortality independent of objective SEP measures likely because it captures facets of socioeconomic position that no objective SEP measure does. Nevertheless, our findings suggest that SSS is partially associated with mortality as a product of wealth.

The implications of our work for public health are considerable. Our findings contribute to a better understanding of socioeconomic inequalities in health and expand the knowledge basis for prevention strategies aiming to reduce socioeconomic inequalities in health. It is important to know that feelings of disadvantage and low social status may lead to increased mortality on the top of the pernicious effect of material disadvantage. This knowledge can be used to fine-tune prevention strategies so that they include empowerment as an additional target next to the main ones of alleviation of material disadvantage and reduction of socioeconomic inequalities in health. Our findings also highlight the existence of important socioeconomic inequalities in people aged ≥ 65, which need to be targeted by prevention strategies, and point out the need to take into account age differences when designing prevention strategies to tackle socioeconomic inequalities in health in adult population.

## Electronic supplementary material

Below is the link to the electronic supplementary material.

Supplementary material 1 (DOCX 162 kb)
