# Supplementary material for: Subjective social status and mortality: the English Longitudinal Study of Ageing
Source: Eur J Epidemiol. 2018 May 19;33(8):729–39. doi: 10.1007/s10654-018-0410-z (PMC6061136; doi:10.1007/s10654-018-0410-z)
Supplement: Supplementary file 1 — Supplementary material 1 (DOCX 162 kb) [file 10654_2018_410_MOESM1_ESM.docx]

**Panayotes Demakakos^1^ • Jane P Biddulph^1^ • Cesar de Oliveira^1^ • Georgios Tsakos^1^ • Michael G Marmot^1^**

**Subjective social status and mortality: the English Longitudinal Study of Ageing**

**^1^Department of Epidemiology and Public Health, University College London, London, UK**

**Panayotes Demakakos, email: p.demakakos@ucl.ac.uk, tel: +44 20 7679 1712**

**--**

**Supplemental File – Online Appendix**

Figure S1 presents the distribution of subjective social status at baseline in participants aged 50 to 64 years. The sample used to create this graph is the original sample of 9312 men and women aged ≥50 years prior to imputing 660 missing at random SSS values.

Figure S2 presents the distribution of subjective social status at baseline in participants aged ≥65 years. The sample used to create this graph is the original sample of 9312 men and women aged ≥50 years prior to imputing 660 missing at random SSS values.

Table S1 presents the descriptive statistics for subjective social status at baseline by age group. The sample used to create this graph is the original sample of 9312 men and women aged ≥50 years prior to imputing 660 missing at random SSS values.

Table S2 presents the associations between subjective social status and all-cause and cause-specific mortality in the original sample of 9312 men and women aged ≥50 years prior to imputing 660 missing at random SSS values.


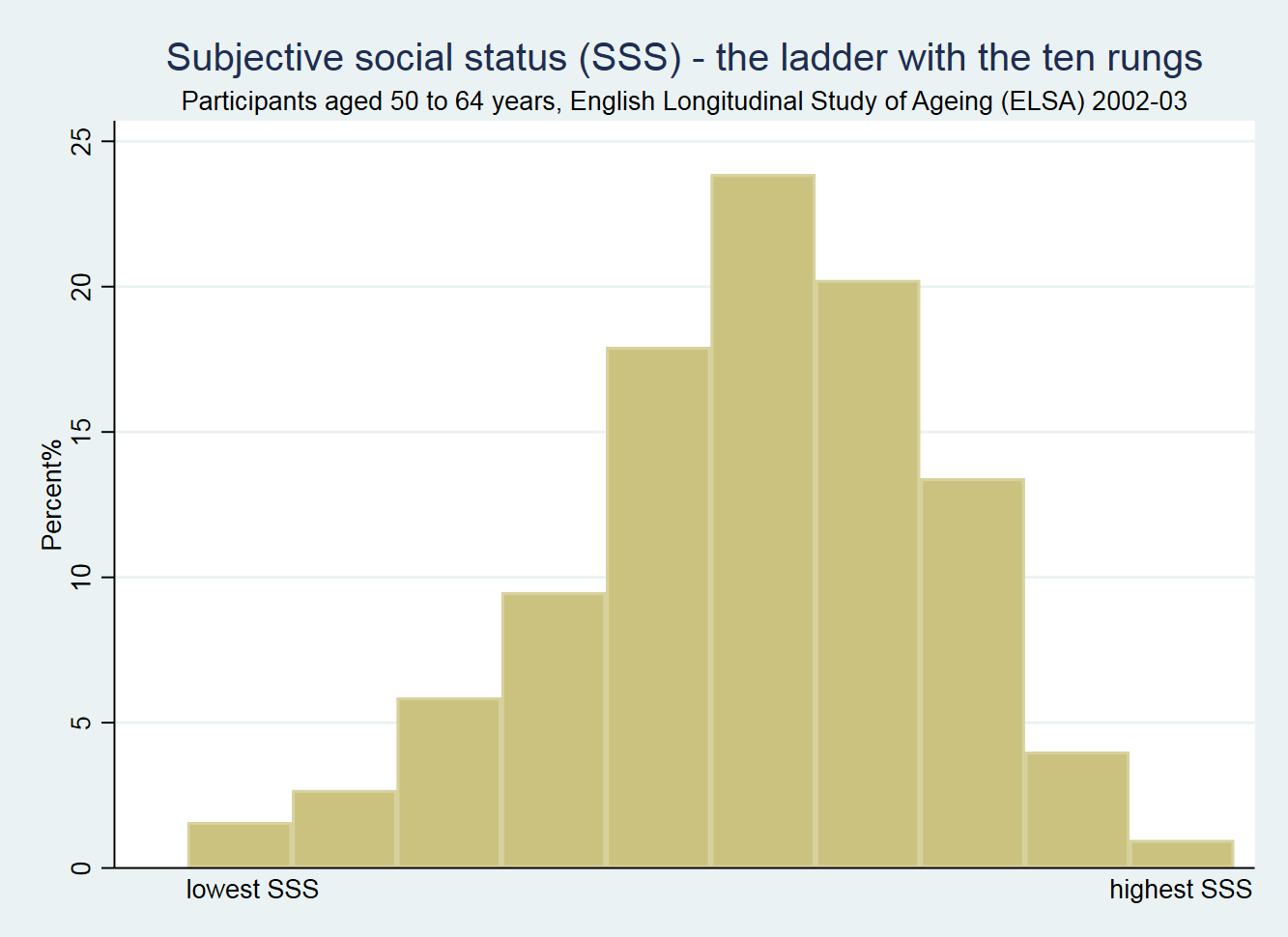


Figure S1. The distribution of subjective social status in participants aged 50 to 64 years – English Longitudinal Study of Ageing (ELSA) 2002-03.


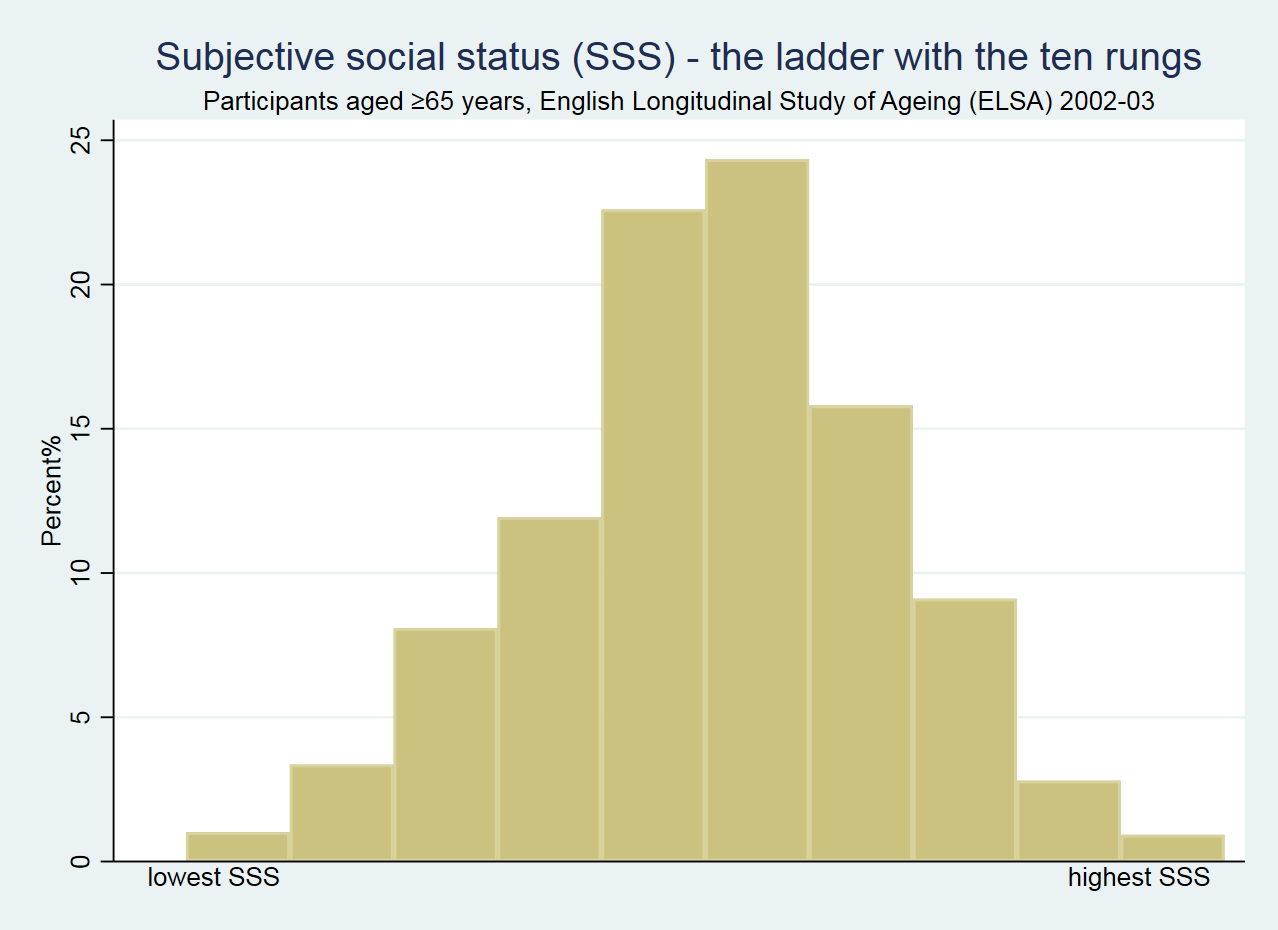


Figure S2. The distribution of subjective social status in participants aged ≥65 years – English Longitudinal Study of Ageing (ELSA) 2002-03

| **Table S1: Subjective social status^a^: the descriptive statistics by age, English Longitudinal Study of Ageing (ELSA) 2002-2003** | | | |
| --- | --- | --- | --- |
|  |  |  |  |
|  | **Age group: 50-64 years** |  | **Age group: ≥65 years** |
| Number of participants | 4998 |  | 4314 |
| Mean SSS (Standard Deviation) | 5.9 (1.8) |  | 5.6 (1.7) |
| 25^th^ centile | 5 |  | 5 |
| Median SSS | 6 |  | 6 |
| 75^th^ centile | 7 |  | 7 |
| ^a^ Subjective social status has not been reversed – higher values denote higher SSS. | | | |

| **Table S2: The associations between subjective social status and all-cause and cause-specific mortality by age,** **English Longitudinal Study of Ageing 2002-2013^a^** | | | |
| --- | --- | --- | --- |
|  |  |  |  |
|  | **Age group: 50-64 years** |  | **Age group: ≥65 years** |
| **All-cause mortality** |  |  |  |
| No of deaths | 373 |  | 1636 |
| Deaths / 1000 person years | 7.4 (6.7-8.1) |  | 43.7 (41.6-45.8) |
| Model 1 HR (95% CI)^a^ | 1.25 (1.18-1.32) |  | 1.07 (1.04-1.10) |
| Model 2 HR (95% CI)^b^ | 1.24 (1.17-1.31) |  | 1.07 (1.04-1.10) |
| Model 3 HR (95% CI)^c^ | 1.12 (1.05-1.18) |  | 1.03 (1.00-1.06) |
| **Cardiovascular mortality** |  |  |  |
| No of deaths | 86 |  | 578 |
| Deaths / 1000 person years | 1.7 (1.4-2.1) |  | 15.4 (14.2-16.7) |
| Model 1 HR (95% CI)^a^ | 1.37 (1.23-1.53) |  | 1.10 (1.05-1.15) |
| Model 2 HR (95% CI)^b^ | 1.38 (1.23-1.54) |  | 1.10 (1.05-1.16) |
| Model 3 HR (95% CI)^c^ | 1.16 (1.03-1.30) |  | 1.06 (1.01-1.11) |
| **Cancer mortality** |  |  |  |
| No of deaths | 186 |  | 466 |
| Deaths / 1000 person years | 3.7 (3.2-4.2) |  | 12.4 (11.4-13.6) |
| Model 1 HR (95% CI)^a^ | 1.13 (1.05-1.22) |  | 1.05 (1.00-1.11) |
| Model 2 HR (95% CI)^b^ | 1.13 (1.05-1.23) |  | 1.06 (1.00-1.11) |
| Model 3 HR (95% CI)^c^ | 1.05 (0.97-1.14) |  | 1.02 (0.97-1.08) |
| **Other mortality** |  |  |  |
| No of deaths | 101 |  | 592 |
| Deaths / 1000 person years | 2.0 (1.6-2.4) |  | 15.8 (14.8-17.2) |
| Model 1 HR (95% CI)^a^ | 1.36 (1.23-1.51) |  | 1.06 (1.01-1.11) |
| Model 2 HR (95% CI)^b^ | 1.34 (1.21-1.48) |  | 1.06 (1.01-1.11) |
| Model 3 HR (95% CI)^c^ | 1.19 (1.07-1.33) |  | 1.00 (0.96-1.05) |
| ***Sample sizes*** |  |  |  |
| No of participants | 4998 |  | 4314 |
| Person years of follow-up | 50700 |  | 37481 |
| HR = Hazard ratio; CI = Confidence interval  Model 1 represents the unadjusted association  Model 2 is adjusted for age, sex, and marital status  Model 3 is adjusted for age, sex, marital status, smoking, physical activity, BMI and elevated depressive symptoms  ^a^Hazard ratios denote hazard change per unit decrease in SSS | | | |
